# Himalayan-Tibetan Plateau Uplift Drives Divergence of Polyploid Poppies: *Meconopsis* Viguier (Papaveraceae)

**DOI:** 10.1371/journal.pone.0099177

**Published:** 2014-06-16

**Authors:** Hongyan Xie, Julian E. Ash, Celeste C. Linde, Saul Cunningham, Adrienne Nicotra

**Affiliations:** 1 Division of Evolution, Ecology and Genetics, Research School of Biology, Australian National University, Canberra, Australian Capital Territory, Australia; 2 Kunming Institute of Botany, Chinese Academy of Sciences, Kunming, Yunnan, People's Republic of China; 3 Ecosystem Sciences, Commonwealth Scientific and Industrial Research Organization, Canberra, Australian Capital Territory, Australia; University of Leicester, United Kingdom

## Abstract

*Meconopsis* Viguier (Papaveraceae) is an iconic genus of alpine forbs that includes medicinal and ornamental species. This study extends previous phylogenetic analyses of *Meconopsis*, using *ITS* sequences representing all the major *Meconopsis* clades. Phenotypic traits are also analysed for all described species. Our results show that *Meconopsis* evolved as a ≥ octaploid clade, with considerable interior structure reflecting further changes in ploidy levels as well as phenotypic differentiation. We support the exclusion of a few species as *Cathcartia* or *Papaver*, making *Meconopsis* a Tibetan region clade. Based on average rates of nucleotide substitution in angiosperm herbs we estimate that the *Meconopsis* clade diverged from the *Meconella* clade of *Papaver* approximately 16.6 Ma. This is soon after the ‘hard’ collision of the Indian continent with Asia caused uplift of the Himalaya and Hengduan ranges, greatly extended the Tibetan plateau, and initiated monsoonal climates. Eight major clades within *Meconopsis* are well supported and these correspond closely to previously recognised subgenus groups. However, the relationship among the clades is poorly resolved, probably because they diverged rapidly ∼15-11 Ma. Two of these clades are ∼dodecaploid but appear to have originated independently. The eight clades have distinct distributions, variously associated with the Himalaya, the eastern Plateau and Hengduan ranges. Some *Meconopsis* species were not monophyletic, suggesting that a combination of multilocus molecular and phenotypic traits is required when defining and revising species.

## Introduction

Many studies suggest that mountain uplift can drive the evolution of new species including plants [1], [2], [3] fungi [4], invertebrates [5], fish [6], frogs [7], birds [8], [9] and mammals [10], [11], [12], [13]. Tertiary uplift of the Tibetan region [14] created the world's largest plateau and highest mountains (Map S1) so evidence of impacts of uplift upon phylogeny should be strong. Uplift increases the diversity of habitats to which species may adapt, extend their range and speciate. Local uplift generally creates cooler, cloudier and wetter habitat but extensive uplift modifies regional climates. Across the Himalo-Tibetan region, hyper-humid monsoonal forests and alpine herb-fields in the south graduate to desert and steppe at similar altitudes to the north [15]. Mountain ranges and plateau margins become deeply eroded creating steep topography with contrasting elevations and aspects that provide high local habitat diversity [16], [17]. Steep altitudinal gradients and complex topography can buffer the impacts of climate change on populations by reducing the need for long-distance migration [18], [19]. However, mountainous terrain also generates barriers that could foster allopatric speciation [20]. In combination, these impacts of mountain uplift on habitat diversity, distribution and stability should have facilitated divergence and speciation in many clades in the Tibetan region. Indications that this occurred are the very high floristic diversity across the southern Tibetan region and phylogenetic evidence from various plant and animal clades [13], [21], [22], [23], [24], [25].

Polyploidization can facilitate a host of rapid evolutionary changes that enable species to exploit the diverse habitats created by mountain uplift [26], [27]. Polyploidization is one of the most effective mechanisms of generating radically new genomes and of causing abrupt speciation [28], [29], [30], [31]. Hybridization introduces an additional genome that can fuel adaptation and divergence. Though autopolyploidy does not add novel genetic material it can cause immediate adaptive changes in phenotype, such as breakdown of self-incompatibility mechanisms [29], [32], [33], [34], as well as providing duplicate copies of genes that can diverge fuelling gradual adaptation [35], [36], [37], [38].

In this paper we focus on *Meconopsis* Viguier (Papaveraceae Jussieu) and examine whether its phylogeny is consistent with an uplift driven model of divergence and speciation. We selected *Meconopsis*, the ‘Tibetan’, ‘Himalayan’ or ‘Blue’ poppies, because the genus is species rich and has been the flagship for the regional flora since the mid-19^th^ century [39], [40], [41], [42]. In 1814 Viguier proposed that the elongate pistil and absence of a protruding stigmatic disc on the capsule of the European *Papaver cambrica* L. warranted recognizing it as a new monotypic genus, *Meconopsis*. Since then more than 70 species with similar traits have been classified as *Meconopsis* [39], [40], [41], [42], [43], [44], [45], [46], [47], [48], [49], [50], [51], [52], [53]. However, taxonomic revisions [39], [52], [53] indicated that these diagnostic traits are not limited to *Meconopsis*. Both the single American and European species have been placed in the *Papaver* clade [52], [54], [55] and one Asian species in *Cathcartia* [39]. The remaining ∼68 *Meconopsis* species are endemic to relatively humid alpine or sub-alpine parts of the Tibetan uplift region (Tibet plateau, Himalaya, Hengduan and adjacent ranges: Map 1). Most species are monocarpic perennial rosette forbs but a few can be polycarpic and one forms ramets. Successive morphological sub-generic classifications [52], [53], [56] have each defined about eight species groups, some of which are consistently identified but others are not, and their proposed hierarchical or evolutionary relationships differ because Prain [52] gave priority to hair shape, Taylor [53] to style shape, and Wu and Chuang [56], [57] to the inflorescence (scapose, racemose or paniculate). Recent collecting has resulted in many new species [43], [44], [45], [46], [47], [48], [49], [50], [51] but some are based on minor morphological differences so there is a need for better phylogenetic information.

DNA sequence analyses are providing a more robust understanding of the phylogeny of the Papaveraceae. The family is a monophyletic clade [58] that fossil evidence indicates diverged from other Ranunculales 121-106 Ma (million years ago) [59] or possibly slightly earlier [60]. An analysis of *ITS* and *trnl* sequences in selected *Papaver*-related species [55] placed Asian *Meconopsis* species as a monophyletic clade that was sister to the *Meconella* clade, the Arctic Poppies (not to be confused with *Meconella* Nuttall the American Fairy Poppies). These two clades combined (*Papaver* 1) are sister to most other *Papaver* species (*Papaver* 2). Carolan *et al* [55] and Yang *et al* [61] included too few *Meconopsis* species to adequately define relationships within the genus.

In this paper we developed an *ITS* phylogeny to investigate how the evolution of *Meconopsis* species might have been influenced by the uplift of eastern Tibetan Plateau and Hengduan ranges. We use the average rate of nucleotide substitution in herbaceous angiosperms [62] to place the *ITS* phylogeny onto a time scale. We also present a phylogeny based on phenotypic traits for all described *Meconopsis* species since this could broaden the analysis to test correlated evolutionary changes in various traits. We map chromosome numbers onto the trees to investigate the possible role of polyploidization in the evolution of *Meconopsis*. Lastly we explore the evolutionary implications of this in the discussion.

## Materials and Methods

### DNA extraction, PCR amplification, cloning and sequencing

We used 75 *Meconopsis*, *Papaver* and *Argemone mexicana* (outgroup) ITS sequences lodged on GenBank and sequenced 34 additional specimens representing 7 *Meconopsis* species that we collected in the eastern Tibetan area (Table S1). The only protected species, *M. torquata*, was donated from harvests made by the Tibetan Traditional Medicine Pharmaceutical Factory, Lhasa, Tibet Autonomous Region. *Meconopsis speciosa* was collected from a wild population (N 28.384014, E 98.993400) with permission from Bai Ma Xue Shan Natural Reserve Bureau. The other species are not protected and permits were not required at the sites where they occurred.

One to 2 g of dried leaf sample was lyophilized and homogenized on a FastPrep machine (Thermo Electron Corp., Milford, MA, USA) for DNA extraction. Genomic DNA was extracted using a DNeasy plant mini kit (QIAGEN Inc., Valencia, CA, USA) following the manufacturer's instructions. DNA extracts were quantified and visualized using agarose gel electrophoresis. *ITS* was amplified using primers *ITS1*+*ITS4* [63] in an Eppendorf Mastercycler. If necessary, PCR bands of interest were excised from the gel and purified using Wizard SV Gel and PCR Clean-up system (Promega, Madison, WI, USA). Purified PCR products from each sample were cloned using the pGEM-T Easy Vector System (Promega). A total of 55 clones representing seven species were sequenced and analyzed. Prior to sequencing, all PCR products were purified using the Promega Wizard SV gel and PCR Clean-up System and ExoSAP-IT (GE Healthcare, Piscataway, New Jersey, USA) according to the manufacturer's instructions. Extension products were purified using an ethanol/EDTA/sodium acetate precipitation protocol according to the BigDye Terminator v3.1 sequencing kit instructions (Applied Biosystems, Foster City, CA, USA). Products were sequenced bi-directionally with ABI PRISM BigDye Terminator v3.1 sequencing kit (Applied Biosystems) on an ABI-3100 automated sequencer. Sequences were edited in Sequencher v 4.7 [64].

### Phylogenetic reconstruction and divergence time estimation

We conducted BLAST [65] searches on the consensus sequences of the obtained *Meconopsis* and related taxa and included the top hits in the phylogenetic analysis. The multiple sequence alignment was conducted with MUSCLE in Geneious Pro v5.6.3 [66] using default settings, and manually optimised. Indels as well as a 12 bp and 17 bp hypervariable region were excluded from the final alignment, similar to Carolan *et al.* [55].

A maximum-likelihood (ML) tree was estimated using RAxML 7.0.4 [67]. We estimated the phylogeny and chronology of clade divergence using BEAUti v1.7.4, BEAST v1.7.4, Tracer v1.5 and TreeAnnotator v1.7.4 [68] to derive a maximum-credibility (MC) tree from the aligned *ITS* data. Trees were visualised using FigTree v1.3.1 [69]. For the MC tree we used a GTR nucleotide substitution model with 4 gamma categories, a Yule speciation process, and a lognormal relaxed clock that allows rates to vary independently among lineages. We set priors for the clock using the mean (4.13×10^−9^ site^−1^ year^−1^) and standard deviation (1.47×10^−9^ site^−1^ year^−1^) of *ITS* nucleotide substitution rates among 10 herbaceous angiosperm clades with calibration ages >0.5 Ma [62]. We used default settings for other priors and ran the Markov chain Monte Carlo algorithm for 2.5×10^7^ generations, discarded the initial 10% as ‘pre-equilibrium’, and then sampled every 2500^th^ generation to derive a consensus tree. The effective sample sizes exceeded 1000 for all summary statistics, which exceeds the threshold of 200 that is considered to be adequate [68]. Support for nodes in topologies was assessed using non-parametric bootstrapping (ML tree) and posterior probabilities (MC tree).

### Phenotypic traits

We included all 68 well-described *Meconopsis* species and four *Papaver* species (*Meconella* clade) [55] as outgroup to root phenotypic cladistic trees. We also generated a tree that included species placed in the *Cathcartia* clade by *ITS* analyses: *C. villosa, M. chelidonifolia, M. oliveriana and M. smithiana*. Phenotypic traits were chosen that have been used to distinguish species and include measures of root, stem, leaf, inflorescence, flower and fruit (Table S2). We categorized count and size measures into 5–7 classes so that we could represent the variability of each trait within species. Successive size classes had a roughly geometric relationship. This produced 251 binary traits that we scored from live or herbarium specimens, published descriptions and keys [39], [40], [45], [46], [48], [49], [50], [51], [56], [70], [71], [72], [73], [74], [75]. Cladistic analysis was conducted using the heuristic method in *Mesquite* [76] with SPR pruning and regrafting routine to select the minimum tree length. After 2.85×10^6^ rearrangements, 100 trees of equal length were found, and these were summarized as a ‘majority rules’ consensus tree.

Linear Discrimination Analysis (LDA in R [77]) is generally robust on binary data [78] and we used it to examine how well the clades defined by the *ITS* analysis could be predicted as groups from the binary phenotypic data. We used a Classification Tree (in R: [79], [80]) to define a minimal set of phenotypic traits that could discriminate all clades.

## Results

### Phylogenetic reconstruction and divergence time estimation

**Figure 1:**
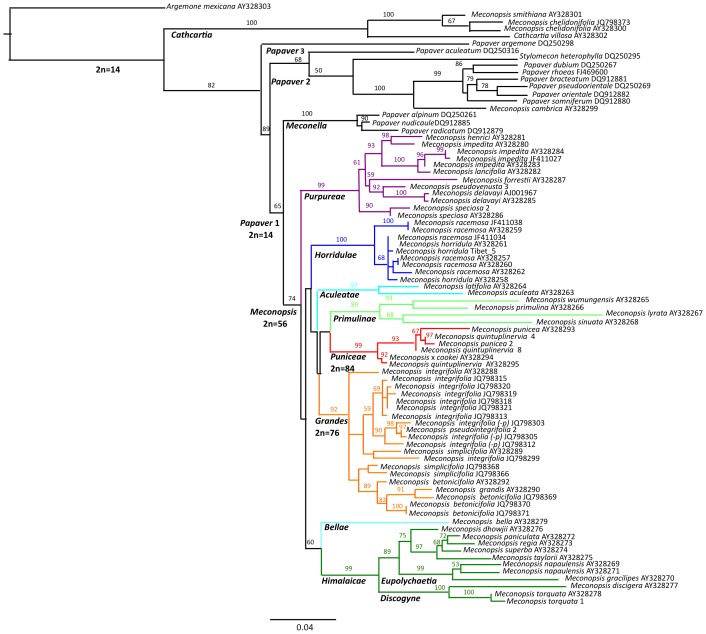
Maximum likelihood (ML) tree based on *ITS* sequences, with *Argemone mexicana* as outgroup. Bootstrap support (%) for nodes is shown. Key nodes are labeled with the inferred diploid (2n) number of chromosomes. Clades are highlighted in colour. Specimens of *M. integrifolia* that we consider could be classified as *M. pseudointegrifolia* are indicated (*p-*). The scale bar represents substitutions per nucleotide site.

**Figure 2:**
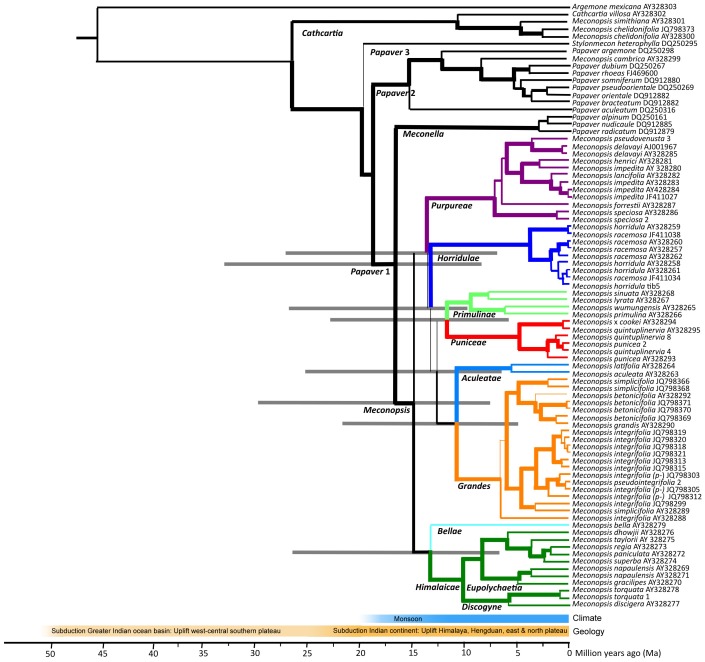
Consensus maximum credibility (MC) tree based on *ITS* sequences, with *Argemone mexicana* as outgroup. Branch thickness indicates bootstrap support (%) for nodes (≥75%, ≥50–75%, ≥25%–50%, <25%). Clades are highlighted by colour. The estimated median ages (***A***) of nodes have approximately lognormal error distributions with 95% confidence intervals of ∼0.5 ***A*** to ∼2 ***A***. 95% error bar for key nodes are indicated by grey lines. Specimens of *M. integrifolia* that we consider could be classified as *M. pseudointegrifolia* are indicated (*p-*). Scale represents median age estimate (Ma).

The ML tree (Fig. 1) reiterates the broad supra-generic phylogeny that Carolan *et al.* [55] found. The majority of *Meconopsis* species are within a monophyletic clade (74% bootstrap support), sister to the Arctic poppies (*Papaver* spp: *Meconella* clade), which together are sister to the *Papaver* 2 clade. The European *M. cambrica* (syn. *P. cambrica*) is placed in *Papaver* 2, and Californian *Stylomecon heterophylla* (syn. *M. heterophylla*) in *Papaver* 3, while Tibetan region *M. chelidonifolia* and *M. smithiana* (syn. *Cathcartia smithiana*) are placed in the *Cathcartia* clade, sister to *Argemone* (67%) in this analysis. Within the *Meconopsis* clade there are eight well supported clades on the Tibetan plateau and adjacent ranges (Fig. 2): *Purpureae* (99%), *Horridulae* (100%), *Aculeatae* (97%), *Primulinae* (89%), *Puniceae* (99%), *Grandes* (92%), *Himalaicae* (99%) and *Bellae* (*M. bella*). The *Himalaicae* comprises the largely Himalayan *Eupolychaetia* (99%) and *Discogyne* (100%) clades. Most of these clades correspond to and are named after existing taxonomic groups, however the traditional *Aculeatae* series [39], [53] corresponds to three distinct clades: *Aculeatae* (including *M. aculeata*), *Purpureae* (purple-flowered species) and *Horridulae* (including *M. horridula*). The traditional *Simplicifolia* series is split between *Grandes* and *Puniceae* (including *M. punicea*) clades. The *Himalaicae* plus *Bellae* clade (80%) is identified as sister clade (60%) to all other *Meconopsis* clades, however other clade nodes are less certain.

Key nodes (Fig. 1) are labeled with the inferred diploid (2n) number of chromosomes [81]. Ploidy is estimated with a base of x = 7. The crown node of the *Papavereae* is diploid with 2n = 2x = 14, whereas the crown of *Meconopsis* is 2n = 8x = 56, the *Puniceae* clade is 2n = 12x = 84, and the crown of the *Grandes* clade has 2n<12x = 76–80 chromosomes, which possibly indicates a chromosome loss from an x = 12 genome.

The consensus maximum credibility (MC) tree (Fig. 2) is broadly similar to the ML tree. The major difference is among the poorly supported nodes (<50% ML bootstrap or <50% MC posterior probability) in the *Meconopsis* clade. Key median crown ages are 18.8 Ma for *Papaver* 1 and *Papaver* 2, 16.6 Ma for *Meconopsis* and *Meconella* clades, 15.0 Ma for *Meconopsis*. All the major *Meconopsis* sub-clades had stem ages >12.5 Ma, and crown ages were: *Purpureae* 7.2 Ma, *Bellae* <13.2 Ma, *Himalaicae* 10.0 Ma, *Eupolychaetia* 8.2 Ma, *Discogyne* 5.6 Ma, *Horridulae* 3.7 Ma, *Primulinae* 9.2 Ma, *Puniceae* 4.7 Ma, and *Grandes* 6.6 Ma.

### Phenotypic tree

**Figure 3:**
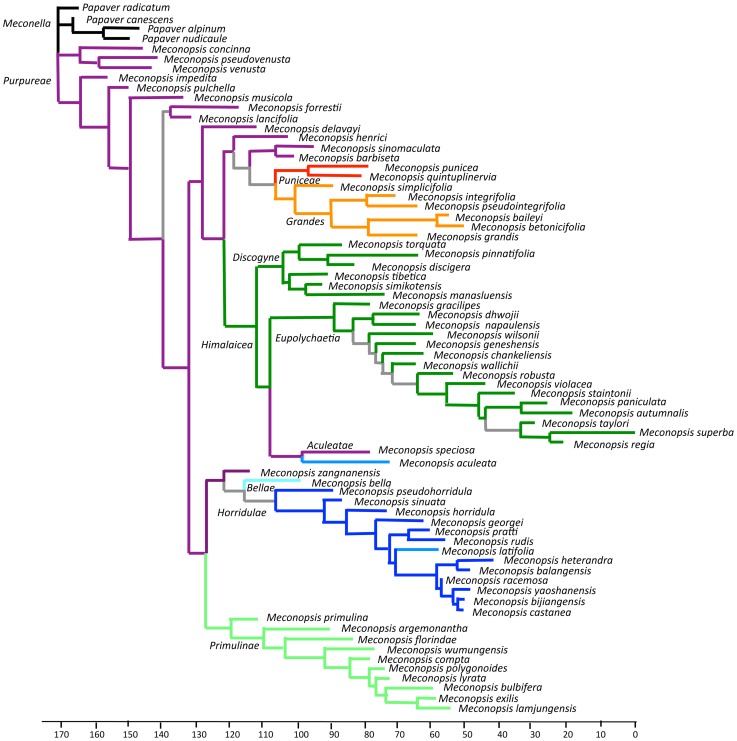
Consensus phenotypic tree for all *Meconopsis* species with selected *Papaver* (*Meconella* clade) species as outgroup based on 251 binary traits for 68 taxa. All other nodes had ≥62% and most had ≥99% support. Colours indicate clades identified on *ITS* trees except grey lines indicate nodes with <50% support. Scale represents branch lengths.

The consensus phenotypic tree for all *Meconopsis* species (Fig. 3) had ≥62% for all nodes and ≥99% support for most nodes. The topology is significantly different from the *ITS* trees. The base of the tree was a polychotomy including the outgroup *Meconella* clade (*Papaver*) and two clades of *Purpureae* species. One *Purpureae* clade gave rise to all other clades, most of which correspond to named *ITS* clades. However, *M. aculeata* (*Aculeatae*) is sister to *M. speciosa* (*Purpureae*) within the *Himalaicae*, and *M. bella* (*Bellae*) is placed as sister to *Horridulae*. On this tree shared basal traits included a scapose inflorescence, 4–6 petals, lobed or dissected leaves, and the relatively small size of most organs. Derived traits included racemose and paniculate inflorescences, larger organ size and more floral parts. When the paniculate *ITS Cathcartia* clade was included in the analysis it was placed within the *Eupolychaetia* clade.

**Figure 4:**
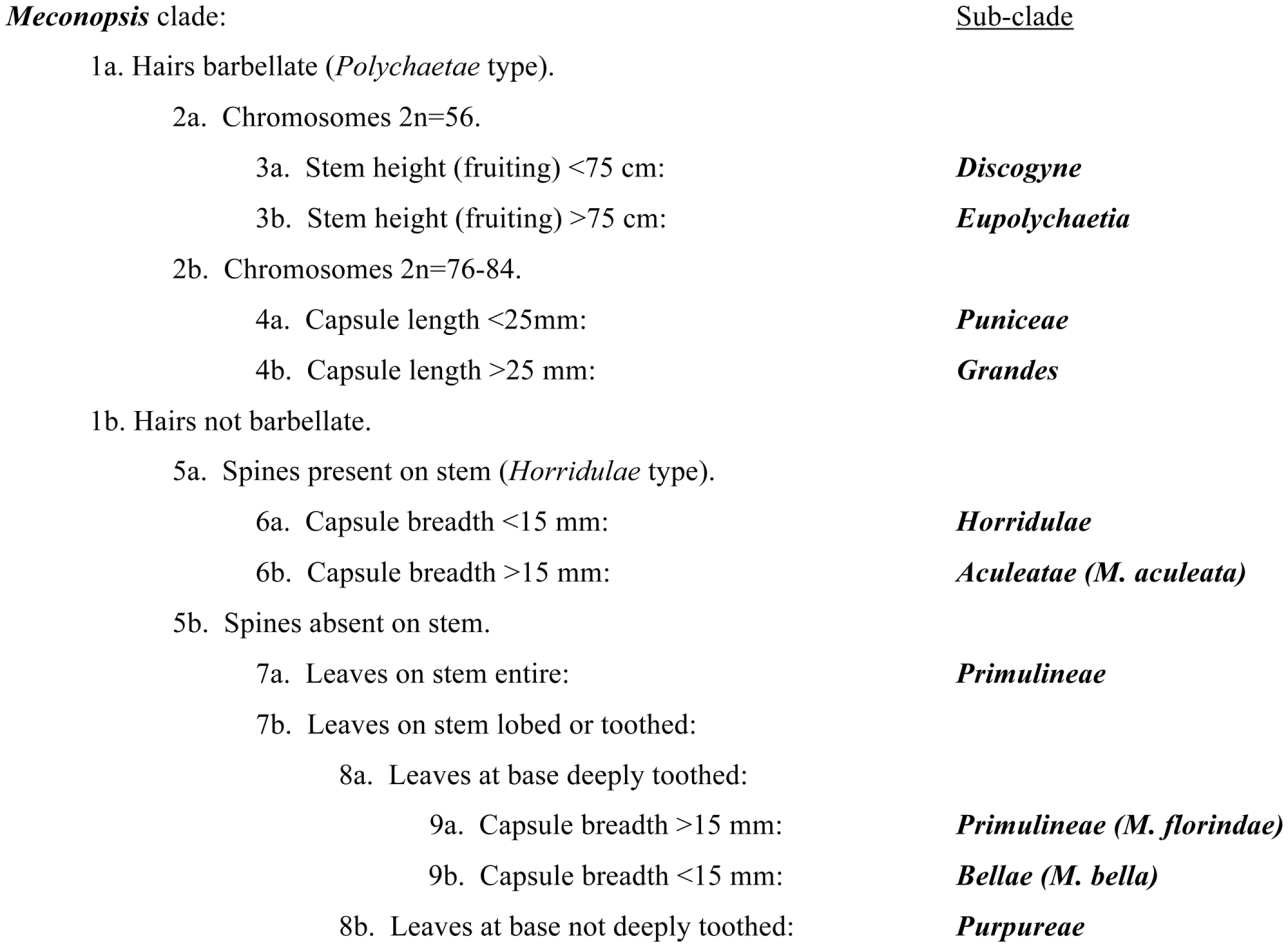
A simple classification tree for *Meconopsis ITS* sub-clades derived from all binary phenotypic data. The tree was found using the Tree Classification routine in R, and is one of several trees of similar length.

The LDA analysis required exclusion of 2 clades represented by a single species and also exclusion of phenotypic traits unique to a single *ITS* clade (potentially the best predictors) however from the remainder it correctly assigned all other species to *ITS* clades. The minimal Classification Tree (Fig. 4) required 9 nodes and 8 traits to define the 9 *ITS* clades, with *M. florindae* separated from other *Primulinae*. Other trees of similar length were found, using different trait combinations.

## Discussion

Our study extends previous phylogenetic studies that included *Meconopsis* species [55], [61] and confirms that *Meconopsis* is monophyletic with considerable interior structure reflecting changes in ploidy levels as well as gradual differentiation. Certain differences are clear between the molecular and phenotypic trees, indicating sources of confusion in previous work and suggesting parallel evolution of certain traits. Here we assess the differences between molecular and phenotypic trees, examine the role of polyploidy, and interpret our results into the context of uplift of the Tibetan region. Lastly we discuss problems associated with delineating species and modes of speciation.

### Molecular and Phenotypic trees

The *ITS* phylogeny reveals several contrasts to the trees based on phenotypic data alone. Notably, the *ITS* phylogeny places the *Cathcartia* clade outside the *Papaver - Meconopsis - Argemone* clade whereas the phenotypic data place it within *Eupolychaetia* (*Meconopsis*), largely due to analogous large shoot and paniculate inflorescence traits. This example highlights the difference: the phenotypic tree suggests that various traits evolved from a small size and few parts to larger size and more parts or branches but this is not supported by the *ITS* trees. Nor is the trend supported if phenotypic traits are examined more widely in the Papaveraceae. Though the *ITS* trees support our choice of *Meconella* clade species as the outgroup to root the phenotypic tree, the *Meconella* clade is adapted to cold arctic and northern alpine habitats where small and simple plants prevail.

We infer that the shared ancestors of the *Meconella* and *Meconopsis* clades had racemose or paniculate inflorescences and that scapes were independently evolved as adaptations to colder and drier habitats. Scapes of high-arctic *Meconella* clade species (e.g. *P. radicatum*) are renowned for heliotropic growth [82] which is absent in *Meconopsis*. The hypothesis that scapes are a derived trait agrees with Chuang [57], however, none of our trees support her proposed phylogeny of the series within *Meconopsis*.

Our LDA and Classification Tree analyses (Fig. 4) indicate that the *Meconopsis* sub-clades defined by *ITS* sequences are also readily defined by phenotypic traits, giving support to their recognition as taxonomic units.

### Abrupt polyploid speciation and the origin of *Meconopsis*

Changes in ploidy level clearly underlie the diversification of *Meconopsis* and some of its constituent clades. We infer that the ancestral chromosome number in the *Papaver* 1 clade was 2n = 2x = 14, since this is found in *Papaver* 2 and the *Meconella* clade of *Papaver* 1 [83], [84]. *Cathcartia* species also have 2n = 2x = 14, supporting the *ITS* phylogeny that places them outside *Papaver*, rather than within *Meconopsis*. Karyotype studies of *Meconopsis* species suggest that all are polyploid (2n = 8–12x = 56–84) [81], [85], [86], [87], but see Kumar *et al.* [88]. We suggest that the *Meconopsis* clade originated with the polyploid transition to 2n = 2x = 56, possibly via a tetraploid intermediate with 2n = 4x = 28 chromosomes that is now extinct. The *ITS* trees suggest that independent polyploid transitions subsequently gave rise to the *Grandes* (2n = 76–80<12x) and *Puniceae* (2n = 12x = 84) clades, however support for this topology is weak. We could not determine if polyploidy in *Meconopsis* was due to autoploidy or alloploidy, and we caution that cladistic methods are not designed to determine the phylogeny of hybrids.

Allopolyploids occur in many clades in the Papaveraceae [83], [89], [90] and autopolyploids are prevalent in the *Meconella* clade [91]. Homoploid hybrids have been induced amongst various related *Meconopsis* species with similar ploidy level [40], [86] and some natural hybrids are known [61], [92].

### Tibetan plateau uplift and the evolution of *Meconopsis*

Placing the evolution of *Meconopsis* into a historical context requires accurate dating. There are reliable dates for the tectonic uplift of the Tibetan region [14], [93], [94], [95], [96], [97] and palaeoclimates [93], [94], [95], [98], [99], [100]. Uplift occurred in two stages [14]. About 52 Ma, the collision and accretion of the Indian Tibetan-Himalaya microcontinent to the Asian plate was followed by subduction of Greater Indian Basin oceanic crust (52 to 25 Ma) and Asian crustal shortening and thickening that caused uplift of the southern west-central Tibetan plateau. About 25-20 Ma Indian continental crust first reached the subduction zone, where upper layers were sheared off and accreted, contributing to the Himalaya, while deeper continental crust was subducted. This ongoing ‘hard’ collision caused uplift of the Himalaya, further uplift and major faulting of the plateau and, from 15-10 Ma, extension of the plateau to the north and east. The Hengduan ranges to the southeast were tilted up from 20-13 Ma, driven largely by deep crustal flow from the plateau [16], [96], [101], [102], [103], [104] (Map S1). The atmospheric pressure system that brings summer monsoonal precipitation and dense cloud cover to the Himalaya, Hengduan ranges and eastern Tibetan plateau was initiated by uplift about 20 Ma [100]. At that time global Tibetan-latitude climates were ∼4°C warmer than at present [105] so alpine climates probably only occurred on elevated parts of the plateau and ranges at 5000 to 6500 m altitude.

Due to a lack of fossils and definitive geological events, our phylogenetic divergence dates were based on the long-term average rate of nucleotide substitution in other angiosperm herbs [106]. This approach has been used in similar studies [61]. We found the median molecular clock dates to be consistent with the hard collision of India with the Tibetan region causing uplift that enabled the early evolution of both *Papaver* 1 and the *Meconopsis* clade.

We suggest that the *Papaver* 1 clade originated in mid- to north- central Asia, since *Meconopsis* is endemic to the Tibetan region and the *Meconella* clade has a center of taxonomic diversity to the north [55], [91], [107]. The uplift driven expansion of subalpine and alpine habitat in this region following the hard collision (starting 25-20 Ma) slightly precedes the median stem and crown ages (18.8 Ma and 16.6 Ma) for *Papaver* 1. The *Meconopsis* and *Meconella* clades include some of the most cold-tolerant angiosperms [108], [109] and we suggest that cold tolerance was an ancestral trait in *Papaver* 1 that enabled proliferation in uplifted regions. Although both clades tolerate extreme winter cold, *Meconopsis* is associated with summer monsoonal climates (that started ∼20 Ma), whereas *Meconella* is not. We suggest that the *Meconella* clade adapted to northern high-latitudes as global climates cooled from ∼15 Ma [110] and only gave rise to the extant clade during the late Cenozoic, with glacial episodes facilitating its wide dispersal [105], [111].

The ranges of most *Meconopsis* sub-clades overlap, and species richness peaks, around the junction of the Himalaya and Hengduan ranges (Map S1 and S2). We propose that *Meconopsis* originated here (∼16.6-15 Ma) soon after uplift commenced (∼20-13 Ma). The transition to tetraploidy (2n = 4x = 28) or octaploidy (2n = 8x = 56) possibly enabled *Meconopsis* to invade cold monsoonal environments. Most well-supported *Meconopsis* sub-clades have similar median stem ages ∼15-12.5 Ma, and poorly resolved ancestral relationships, indicating rapid early divergence. There are both habitat and biogeographic differences amongst the sub-clades. The *Himalaicae* (*Eupolychaetia* and *Discogyne*), *Bellae* and *Primulinae* clades spread west along the monsoonal Himalayan arc, with *Discogyne* adapting to the drier Trans-Himalaya. The ∼dodecaploid *Grandes* clade also occur in the Himalaya and Hengduan ranges but *M. integrifolia* and *M. pseudointegrifolia* have adapted to drier plateau habitats to the north. The *Purpureae* and dodecaploid *Puniceae* clades spread across the eastern plateau and Hengduan ranges. The *Horridulae* clade spread widely into relatively dry and cold habitats: west along the Himalaya and adjacent plateau, along the Hengduan ranges and northeast across the plateau. The *Aculeatae* clade is restricted to the dry western Himalaya, and morphologically appears to be an extension of the *Horridulae* clade.

### Species variability and delineation

A few species were represented by several specimens in our *ITS* trees (Fig. 1 & 2), allowing evaluation of species monophyly. Several species in the *Purpureae* and *Horridulae* clades were not monophyletic but rather form groups (e.g. *M. impedita*, *M. henrici* and *M. lancifolia*; *M. horridula* and *M. racemosa*), indicating either that some specimens were incorrectly identified or that species boundaries require revision. Various *Meconopsis* species are only distinguished by minor morphological trait differences, apparent as short branch lengths on the phenotypic tree (Fig. 3). Analysis of DNA sequence loci on geographically distributed specimens of these and related species could clarify their phylogeny and taxonomic status. Many of these problematic taxa occur within small areas of the eastern Tibetan plateau and Hengduan ranges and may represent geographic differentiation of widespread species. DNA sequences of ‘*M. integrifolia*’ (including *M. pseudointegrifolia*) populations across this region show such local spatial differentiation [61]. From our knowledge of local morphology we assigned these ‘*M. integrifolia*’ populations to either *M. integrifolia* or *M. pseudointegrifolia* [39] and conclude from the phylogenetic trees [61] that these are northern and southern sister clades. A population genetic approach is needed to assess whether these populations represent a single variable species, a cline or separate entities.

We suggest that the complex mosaic of habitats on the eastern Tibetan plateau and northern Hengduan ranges facilitated local population divergence. Minor differences in traits are common amongst these populations of *Meconopsis* species and we expect similar patterns to emerge in other plant genera in this region. Ongoing uplift in the southern Hengduan ranges is raising peaks into the alpine zone facilitating ‘island’ speciation, e.g. *M. delavayi*. In contrast, alpine habitat along the 2400 km Himalayan arc forms a nearly continuous but convoluted (Fractal Dimension ∼1.3 [112], [113], [114]) contour belt averaging ∼4.4 km wide but ∼16000 km long (total area ∼1.4×10^5^ km^2^) (Map S1 and S2). Himalayan *Meconopsis* species are mostly restricted to short portions of the arc, and speciation has possibly occurred by parapatric divergence. *Meconopsis* species diversity declines to the west, which receives lower precipitation, and we suggest that the *Eupolychaetia* and *Primulinae* clades originated in the Eastern Himalaya. These hypotheses could be tested by multilocus sequence loci and population-level DNA analyses.

## Conclusions

We consider the *ITS* phylogeny provides a robust indication of the evolution of *Meconopsis*, whereas the phenotypic is confounded by false homologies and convergent evolution. We used the average and standard deviation of the nucleotide substitution rate in herbaceous angiosperms [61], [106] to date nodes, and we found the median dates follow shortly after geological and palaeoclimate events that created suitable habitat for *Meconopsis*. We support Carolan *et al* [55] in placing *Meconopsis* as sister clade to the Arctic Poppies (*Meconella* clade) and estimate that these diverged about 18.8-16.6 Ma, probably in uplifted areas of central Asia. We consider the transition to tetraploidy or octaploidy (2n = 4x or 8x = 28 or 56) was instrumental in the early evolution of *Meconopsis*. Subsequently two ∼dodecaploid (2n≈12x = 76–84) clades arose within *Meconopsis*, both of which are characterized by robust and abundant species. The relationship among clades within *Meconopsis* is poorly resolved and requires additional sequence information. In contrast, there is strong support for most of clades (*Discogyne*, *Eupolychaetia*, *Bellae* and *Primulinae*), and they correspond closely to existing taxonomic groups. We found that some species in the *Aculeatae*, *Purpureae* and *Horridulae* clades were not monophyletic and suggest that species boundaries require revision. We noted that these species are associated with the eastern Plateau and Hengduan ranges that provide an extensive pattern of habitat variation that could foster a mosaic of locally divergent populations. The charismatic *Meconopsis* group provides an exciting opportunity for further investigation of the role of geological events, ploidy and local adaption in the diversification of high elevation lineages – of which we know relatively little. We suggest that similar analyses of other alpine herbarium genera from this region can test the paleao-biogeographic hypothesis that we have proposed.

## Supporting Information

Table S1 Species, voucher specimens codes and GenBank accession numbers for ITS sequences used in the study.
(DOCX)

Table S2 Binary phenotypic traits of 68 *Meconopsis* species and 4 *Papaver* (*Meconella* clade) species used in the phenotypic analyses.
(XLSX)

Map S1 The Tibetan region and adjoining parts of Indian and Asian tectonic plates, showing the subduction zone and major uplifted topographic regions. The distribution of *Meconopsis* is indicated, including areas with ≥1, ≥5 and ≥10 species. Both *Meconopsis* species and sub-clade richness peak at the junction of the Himalaya and Hengduan ranges. Base map from NASA Terra Image.
(TIF)

Map S2 Map of the ranges of the nine *Meconopsis* clades defined by *ITS* sequences. Ranges are based on herbarium specimens and field observations. Note that species are patchily distributed within these ranges and that sampling is sparse over much of the region. The centre of clade and species diversity is indicated by a star, which could indicate where *Meconopsis* originated. Most clades have 2n = 56 chromosomes. The *Eupolychaetia*, *Discogyne*, *Bellae* and *Primulinae* clades are largely associated with the Himalayan and Hengduan ranges at the southern margin of the Tibetan uplift region. The *Grandes* clade (2n = 76–80) is widely represented on the plateau by *M. integrifolia*. The *Puniceae* clade (2n = 84) apparently evolved on the northeast plateau, and *M. quintuplinervia* has spread further East onto adjacent mountain ranges. The base map from NASA Terra Image.
(TIF)
