# Supplementary material for: Himalayan-Tibetan Plateau Uplift Drives Divergence of Polyploid Poppies: Meconopsis Viguier (Papaveraceae)
Source: PLoS One. 2014 Jun 16;9(6):e99177. doi: 10.1371/journal.pone.0099177 (PMC4059618; doi:10.1371/journal.pone.0099177)
Supplement: Table S1 — Species, voucher specimens codes and GenBank accession numbers for ITS sequences used in the study. (DOCX) [file pone.0099177.s001.docx]

Table S1. Species, voucher specimens codes and GenBank accession numbers for ITS sequences used in the study.

| **Species** | **GenBank Accession number** | **Reference** |
| --- | --- | --- |
| *Argemone mexicana* Linn. (Ghamoya) | AY328303 | [[1](#_ENREF_1)] |
| *Cathcartia (Meconopsis) villosa* Hook.f. ex Hook. | AY328302 | [[1](#_ENREF_1)] |
| *Meconopsis aculeata* Royle | AY328263 | [[1](#_ENREF_1)] |
| *Meconopsis bella* Prain | AY328279 | [[1](#_ENREF_1)] |
| *Meconopsis betonicifolia* Franchet | DQ250323 | [[2](#_ENREF_2)] |
| *Meconopsis betonicifolia* Franchet | AY328292 | [[1](#_ENREF_1)] |
| *Meconopsis betonicifolia* Franchet | JQ798370 | [[3](#_ENREF_3)] |
| *Meconopsis betonicifolia* Franchet | JQ798369 | [[3](#_ENREF_3)] |
| *Meconopsis cambrica* (L.) Vig. | AY328299 | [[1](#_ENREF_1)] |
| *Meconopsis chelidoniifolia* Bureau & Franchet | AY328300 | [[1](#_ENREF_1)] |
| *Meconopsis chelidoniifolia* Bureau & Franchet | JQ798373 | [[3](#_ENREF_3)] |
| *Meconopsis delavayi* (Franchet) Franchet ex Prain | AY328285 | [[1](#_ENREF_1)] |
| *Meconopsis delavayi* (Franchet) Franchet ex Prain | AJ001967 | [[4](#_ENREF_4)] |
| *Meconopsis dhowjii* G. Taylor ex Hay | AY328276 | [[1](#_ENREF_1)] |
| *Meconopsis discigera* Prain | AY328277 | [[1](#_ENREF_1)] |
| *Meconopsis forrestii* Prain | AY328287 | [[1](#_ENREF_1)] |
| *Meconopsis gracilipes* G. Taylor | AY328270 | [[1](#_ENREF_1)] |
| *Meconopsis grandis* Prain | AY328290 | [[1](#_ENREF_1)] |
| *Meconopsis henrici* Bureau & Franchet | AY328281 | [[1](#_ENREF_1)] |
| *Meconopsis horridula* Hooker.f. & Thomson | AY328261 | [[1](#_ENREF_1)] |
| *Meconopsis horridula* Hooker.f. & Thomson | AY328258 | [[1](#_ENREF_1)] |
| *Meconopsis horridula* Hooker.f. & Thomson | Tib 5 |  |
| *Meconopsis impedita* Prain | AY328283 | [[1](#_ENREF_1)] |
| *Meconopsis impedita* Prain | AY328284 | [[1](#_ENREF_1)] |
| *Meconopsis impedita* Prain | AY328280 | [[1](#_ENREF_1)] |
| *Meconopsis impedita* Prain | JF411027 | [[5](#_ENREF_5)] |
| *Meconopsis integrifolia* (Maximowicz) Franchet | AY328288 | [[1](#_ENREF_1)] |
| *Meconopsis integrifolia* (Maximowicz) Franchet | JQ798320 | [[3](#_ENREF_3)] |
| *Meconopsis integrifolia* (Maximowicz) Franchet | JQ798315 | [[3](#_ENREF_3)] |
| *Meconopsis integrifolia* (Maximowicz) Franchet | JQ798321 | [[3](#_ENREF_3)] |
| *Meconopsis integrifolia* (Maximowicz) Franchet | JQ798318 | [[3](#_ENREF_3)] |
| *Meconopsis integrifolia* (Maximowicz) Franchet | JQ798319 | [[3](#_ENREF_3)] |
| *Meconopsis integrifolia (pseudointegrifolia)* Prain | Zhd2006.18 |  |
| *Meconopsis integrifolia (pseudointegrifolia)* Prain | JQ798303 | [[3](#_ENREF_3)] |
| *Meconopsis integrifolia (pseudointegrifolia)* Prain | JQ798313 | [[3](#_ENREF_3)] |
| *Meconopsis integrifolia (pseudointegrifolia)* Prain | JQ798305 | [[3](#_ENREF_3)] |
| *Meconopsis integrifolia (pseudointegrifolia)* Prain | JQ798312 | [[3](#_ENREF_3)] |
| *Meconopsis integrifolia (pseudointegrifolia)* Prain | JQ798299 | [[3](#_ENREF_3)] |
| *Meconopsis lancifolia* (Franchet) Franchet ex Prain | AY328282 | [[1](#_ENREF_1)] |
| [*Meconopsis latifolia* (Prain) Prain](#RANGE!_ENREF_10) | AY328264 | [[1](#_ENREF_1)] |
| *Meconopsis lyrata* (H. A. Cummins & Prain) Fedde ex Prain | AY328267 | [[1](#_ENREF_1)] |
| *Meconopsis napaulensis* DC. | AY328271 | [[1](#_ENREF_1)] |
| *Meconopsis napaulensis* DC. | AY328269 | [[1](#_ENREF_1)] |
| *Meconopsis paniculata* (D. Don) Prain | AY328272 | [[1](#_ENREF_1)] |
| *Meconopsis primulina* Prain | AY328266 | [[1](#_ENREF_1)] |
| *Meconopsis pseudovenusta* Prain | Zhd2006.37 |  |
| *Meconopsis punicea* Maximowicz | AY328293 | [[1](#_ENREF_1)] |
| *Meconopsis punicea* Maximowicz | 2007.68 |  |
| *Meconopsis quintuplinervia* Regel | AY328295 | [[1](#_ENREF_1)] |
| *Meconopsis quintuplinervia* Regel | 4 (2007.78) |  |
| *Meconopsis quintuplinervia* Regel | 8( 2008.86) |  |
| *Meconopsis racemosa* Maximowicz | AY328262 | [[1](#_ENREF_1)] |
| *Meconopsis racemosa* Maximowicz | AY328260 | [[1](#_ENREF_1)] |
| *Meconopsis racemosa* Maximowicz | AY328259 | [[1](#_ENREF_1)] |
| *Meconopsis racemosa* Maximowicz | AY328257 | [[1](#_ENREF_1)] |
| *Meconopsis racemosa* Maximowicz | FJ411034 | [[5](#_ENREF_5)] |
| *Meconopsis regia* G. Taylor | AY328273 | [[1](#_ENREF_1)] |
| *Meconopsis simplicifolia* (D. Don) Walpers | AY328289 | [[1](#_ENREF_1)] |
| *Meconopsis simplicifolia* (D. Don) Walpers | JQ 798368 | [[3](#_ENREF_3)] |
| *Meconopsis simplicifolia* (D. Don) Walpers | JQ 798366 | [[3](#_ENREF_3)] |
| *Meconopsis sinuta* Prain | AY328268 | [[1](#_ENREF_1)] |
| *Meconopsis smithiana* (Handel-Mazzetti) G. Taylor ex Handel- Mazzetti, | AY328301 | [[1](#_ENREF_1)] |
| *Meconopsis speciosa* Prain | AY328286 | [[1](#_ENREF_1)] |
| *Meconopsis speciosa* Prain | 2007.25 |  |
| *Meconopsis superb* King ex Prain | AY328274 | [[1](#_ENREF_1)] |
| *Meconopsis taylorii* L.H.J. Williams | AY328275 | [[1](#_ENREF_1)] |
| *Meconopsis torquata* Prain | AY328278 | [[1](#_ENREF_1)] |
| *Meconopsis torquata* Prain | 1 |  |
| *Meconopsis wumungensis* K. M. Feng ex C. Y. Wu & H. Chuang in C. Y. Wu | AY328265 | [[1](#_ENREF_1)] |
| *Meconopsis* x *cookei* | AY328294 | [[1](#_ENREF_1)] |
| *Papaver aculeatum* Thunb. | DQ250316 | [[2](#_ENREF_2)] |
| *Papaver alpinum* L. | DQ250261 | [[2](#_ENREF_2)] |
| *Papaver argemone* L. | DQ250298 | [[2](#_ENREF_2)] |
| *Papaver bracteatum* Lindl | DQ912881 | [[6](#_ENREF_6)] |
| *Papaver bracteatum* Lindl | DQ250287 | [[2](#_ENREF_2)] |
| *Papaver dubium* L. | DQ250267 | [[2](#_ENREF_2)] |
| *Papaver nudicaule* L. | DQ912885 | [[6](#_ENREF_6)] |
| *Papaver orientale* L. | DQ912882 | [[6](#_ENREF_6)] |
| *Papaver pseudoorientale* (Fedde) Medw. | DQ250269 | [[2](#_ENREF_2)] |
| *Papaver radicatum* L. | DQ912879 | [[6](#_ENREF_6)] |
| *Papaver rhoeas* L. | FJ469600 | [[7](#_ENREF_7)] |
| *Papaver somniferum* L. | DQ912880 | [[6](#_ENREF_6)] |
| *Stylomecon heterophylla* G.Taylor | DQ250295 | [[2](#_ENREF_2)] |

References:

1. Yuan C, Shi S, Boufford DE (2006) Phylogenetic analysis of *Meconopsis* Vig. (Papaveraceae) based on chloroplast and nuclear ribosomal DNA sequences. Unpublished manuscript.

2. Carolan JC, Hook ILI, Chase MW, Kadereit JW, Hodkinson TR (2006) Phylogenetics of *Papaver* and related genera based on DNA sequences from ITS nuclear ribosomal DNA and plastid trnL intron and trnL-F intergenic spacers. Annals of Botany 98: 141-155.

3. Yang FS, Qin AL, Wang XQ (2012) Great Genetic Differentiation among Populations of *Meconopsis integrifolia* and Its Implication for Plant Speciation in the Qinghai-Tibetan Plateau Plos One 7: e37196.

4. Blattner FR, Kadereit JW (1999) Morphological evolution and ecological diversification of the forest-dwelling poppies (Papaveraceae : Chelidonioideae) as deduced from a molecular phylogeny of the ITS region. Plant Systematics and Evolution 219: 181-197.

5. Li R, Dao Z (2011) Identification of *Meconopsis* species by a DNA barcode sequence: The nuclear internal transcribed spacer (ITS) region of ribosomal deoxyribonucleic acid (DNA). African Journal of Biotechnology 10: 15805-15807.

6. Lee EJ, Hwang IK, Kim NY, Lee KL, Han MS, et al. (2010) An Assessment of the Utility of Universal and Specific Genetic Markers for Opium Poppy Identification. Journal of Forensic Sciences 55: 1202-1208.

7. Lee EJ, Yang MS (2008) The discrimination study of poppy families. Unpublished manuscript.
