# Supplementary figures and images for: Himalayan-Tibetan Plateau Uplift Drives Divergence of Polyploid Poppies: Meconopsis Viguier (Papaveraceae)
Source: PLoS One. 2014 Jun 16;9(6):e99177. doi: 10.1371/journal.pone.0099177 (PMC4059618; doi:10.1371/journal.pone.0099177)

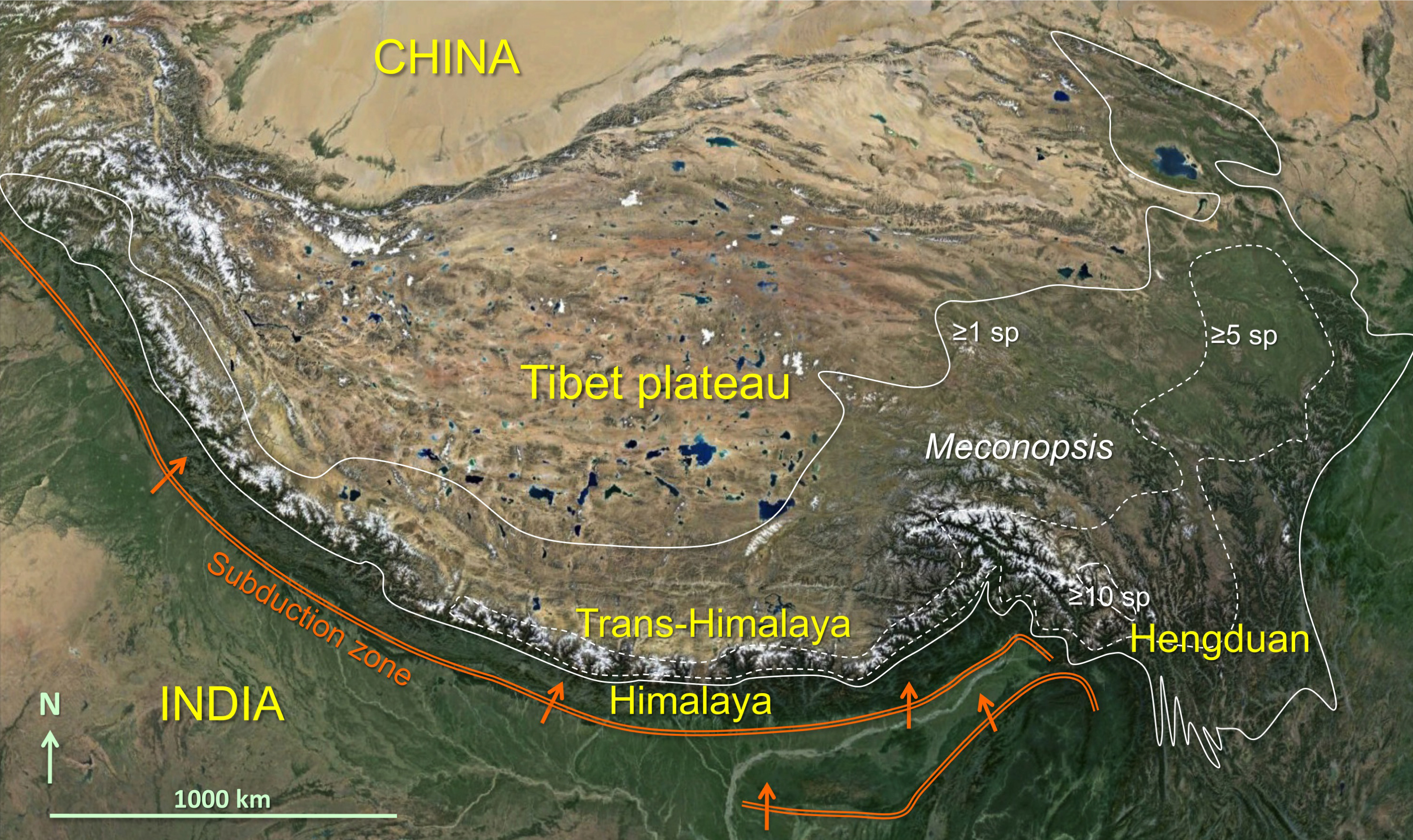

Supplement: Map S1 — The Tibetan region and adjoining parts of Indian and Asian tectonic plates, showing the subduction zone and major uplifted topographic regions. The distribution of Meconopsis is indicated, including areas with ≥1, ≥5 and ≥10 species. Both Meconopsis species and sub-clade richness peak at the junction of the Himalaya and Hengduan ranges. Base map from NASA Terra Image. (TIF) [file pone.0099177.s003.tif]

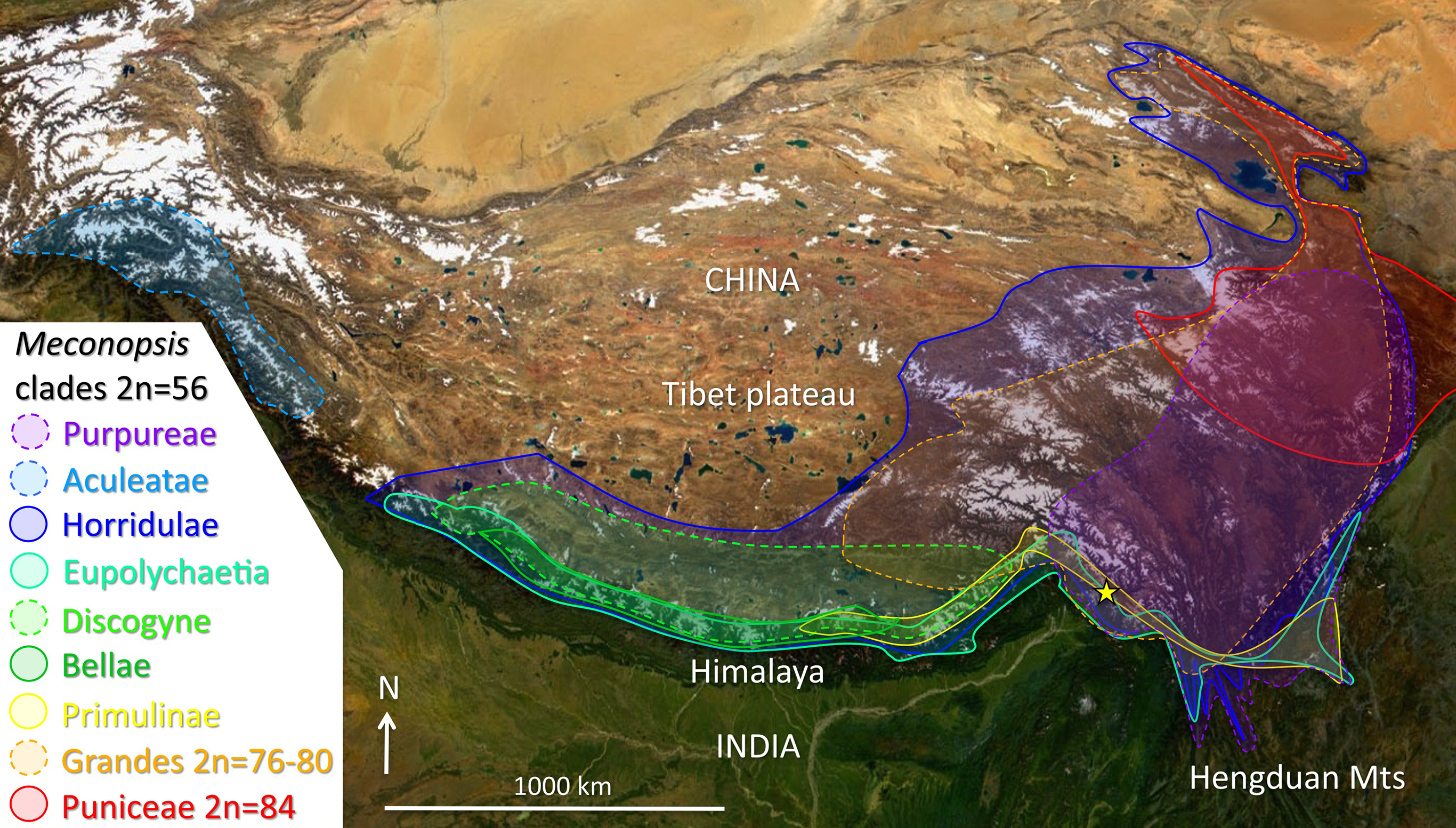

Supplement: Map S2 — Map of the ranges of the nine Meconopsis clades defined by ITS sequences. Ranges are based on herbarium specimens and field observations. Note that species are patchily distributed within these ranges and that sampling is sparse over much of the region. The centre of clade and species diversity is indicated by a star, which could indicate where Meconopsis originated. Most clades have 2n = 56 chromosomes. The Eupolychaetia, Discogyne, Bellae and Primulinae clades are largely associated with the Himalayan and Hengduan ranges at the southern margin of the Tibetan uplift region. The Grandes clade (2n = 76–80) is widely represented on the plateau by M. integrifolia. The Puniceae clade (2n = 84) apparently evolved on the northeast plateau, and M. quintuplinervia has spread further East onto adjacent mountain ranges. The base map from NASA Terra Image. (TIF) [file pone.0099177.s004.tif]
